# AI and Ethics: A Systematic Review of the Ethical Considerations of Large Language Model Use in Surgery Research

**DOI:** 10.3390/healthcare12080825

**Published:** 2024-04-13

**Authors:** Sophia M. Pressman, Sahar Borna, Cesar A. Gomez-Cabello, Syed A. Haider, Clifton Haider, Antonio J. Forte

**Affiliations:** 1 Division of Plastic Surgery, Mayo Clinic, Jacksonville, FL 32224, USA; 2 Department of Physiology and Biomedical Engineering, Mayo Clinic, Rochester, MN 55905, USA; 3 Center for Digital Health, Mayo Clinic, Rochester, MN 55905, USA

**Keywords:** artificial intelligence (AI), ChatGPT, deep learning, machine learning, clinical medicine, surgical specialties, bioethical issues

## Abstract

Introduction: As large language models receive greater attention in medical research, the investigation of ethical considerations is warranted. This review aims to explore surgery literature to identify ethical concerns surrounding these artificial intelligence models and evaluate how autonomy, beneficence, nonmaleficence, and justice are represented within these ethical discussions to provide insights in order to guide further research and practice. Methods: A systematic review was conducted in accordance with the Preferred Reporting Items for Systematic Reviews and Meta-Analyses guidelines. Five electronic databases were searched in October 2023. Eligible studies included surgery-related articles that focused on large language models and contained adequate ethical discussion. Study details, including specialty and ethical concerns, were collected. Results: The literature search yielded 1179 articles, with 53 meeting the inclusion criteria. Plastic surgery, orthopedic surgery, and neurosurgery were the most represented surgical specialties. Autonomy was the most explicitly cited ethical principle. The most frequently discussed ethical concern was accuracy (n = 45, 84.9%), followed by bias, patient confidentiality, and responsibility. Conclusion: The ethical implications of using large language models in surgery are complex and evolving. The integration of these models into surgery necessitates continuous ethical discourse to ensure responsible and ethical use, balancing technological advancement with human dignity and safety.

## 1. Introduction

Artificial intelligence (AI) strives to endow machines with capabilities that are reminiscent of human cognition [1,2]. One type of AI tool in particular, known as a large language model (LLM), has garnered significant attention in recent years. Powered by natural language processing (NLP) and machine learning, LLMs leverage complex neural network architectures [3]. These AI systems are trained on vast quantities of text data, and ultimately develop proficiency in comprehending and generating human-quality text [4]. Released in late 2022, OpenAI’s Chat Generative Pre-Trained Transformer (ChatGPT) [5] is a standout LLM that distinguishes itself through its proficiency in using multiple languages and its capacity to generate nuanced, contextually aware responses. LLMs like ChatGPT show great promise in terms of being useful in healthcare, especially in surgery. The surgical applications of LLMs include assisting in personalized surgical consultations [6,7,8], facilitating the informed consent process [6,9], enhancing patient communication and instructions [8,9,10], and contributing to the education of surgical trainees [8,9,11,12]. Nevertheless, the emergence of these models has stirred considerable debate within the academic and medical community, primarily due to concerns over inherent biases [13,14,15,16], patient data privacy [17,18], and the propagation of misinformation [19]. While AI undoubtedly presents opportunities for advancements, it is imperative to approach its integration with a measured sense of caution, acknowledging the inherent risks and limitations of its use. 

Ethical considerations surrounding LLMs are particularly critical. Engaging in ethical discussions within the scientific community promotes ethical and humane research conduct. In 2019, the High-Level Expert Group (HLEG) presented guidelines for the development of trustworthy AI and demanded that AI respect ethical principles and values [20]. Ethical guidelines for LLMs and their ethical implementation are vital for ensuring human safety; however, achieving a universally accepted framework for these guidelines may prove challenging [18]. In the United States, the predominant approach to biomedical ethics is firmly anchored in the moral theory of principlism, a framework introduced in 1979 by Beauchamp and Childress [21]. Within this framework, four core ethical principles were established: autonomy, beneficence, nonmaleficence, and justice. These core ethical principles are described in Table 1, with corresponding examples. Although important, ethical discussions are not always found within research articles. One study found that surgical literature contains significantly less bioethical content compared to medical literature [22]. 

Despite a paucity in the surgical literature, ethical discussions are an essential component of surgery research for several critical reasons. Firstly, surgery inherently carries risks, making it crucial to ensure that its potential benefits outweigh the potential harms for each individual patient [23]. This involves open and transparent discussions to ensure that informed consent is attained, where patients fully understand the risks and benefits of a procedure before agreeing to participate [23,24]. Secondly, the unique nature of surgery, often involving irreversible procedures [25], demands careful consideration of the ethical implications. Balancing innovation with patient safety requires the thoughtful consideration of acceptable levels of risk in procedures and the pursuit of knowledge when alternatives exist [26]. Thirdly, ethical dilemmas surrounding resource allocation, patient selection, and potential conflicts of interest necessitate continuous analysis and discussion [23,25]. Public trust in surgery research hinges on demonstrating ethical conduct and addressing these complex issues openly. Finally, engaging in ethical discussions fosters a culture of transparency and accountability within the research community, ultimately leading to better research practices and improved patient outcomes [24,27]. By integrating ethical considerations throughout the research process, we can ensure that advancements, such as LLMs and AI, prioritize both scientific progress and the well-being of patients.

With the burgeoning wave of research investigating the implications of LLMs in medicine, we hypothesize that exploring the literature will reveal pervasive ethical concerns. Additionally, specific surgical specialties are likely to present distinct ethical considerations. It is imperative to delve into the ethical considerations, as these will provide invaluable insights with which to guide future research and practice. The purpose of this systematic review is to explore surgery literature to identify the ethical concerns around LLMs using the lens of ethical principlism and evaluate how autonomy, beneficence, nonmaleficence, and justice are represented within these ethical discussions. Furthermore, this review aims to assess the significance of the uncovered ethical concerns in order to provide insights into the current ethical landscape surrounding LLMs in surgery, highlighting areas of concern and potential areas for improvement or mitigation.

## 2. Materials and Methods

**Figure 1:**
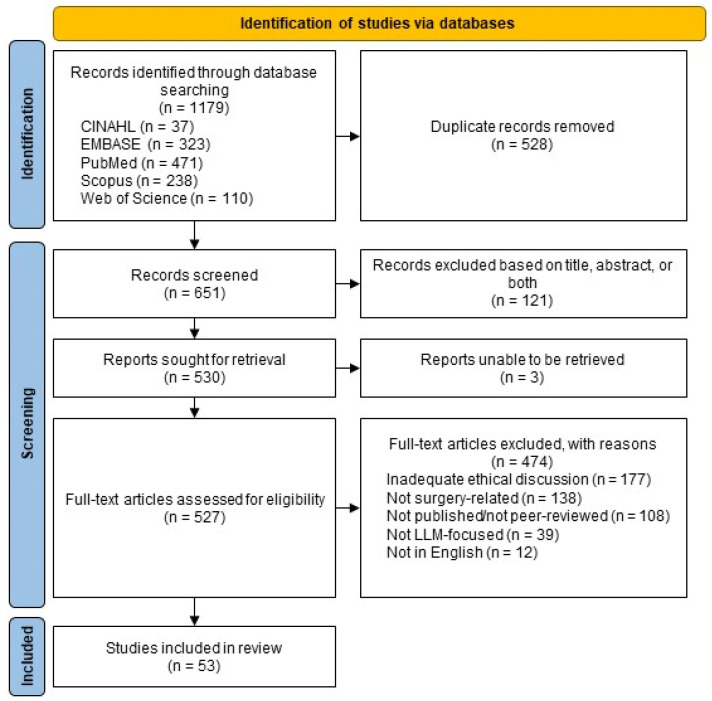
Modified PRISMA 2020 flow diagram for studies included within this systematic review.

This systematic review was conducted following the Preferred Reporting Items for Systematic Reviews and Meta-Analyses (PRISMA) guidelines [28]. These PRISMA guidelines were used to provide an organizational framework for this systematic review and to guide screening and eligibility assessment (Figure 1). A protocol was not registered for this review.

### 2.1. Search Strategy and Database Search

This review concentrated on surgery-related articles that discussed LLMs. In order to optimize the retrieval of relevant articles, a search strategy was developed that used search terms related to LLMs and surgery. Search terms related to LLMs included “large language model*” and terms relating to specific LLMs such as “GPT”. Search terms related to surgery included “surgery”, “surgical”, and “surgeon”, as well as terms related to specific surgical specialties. Appropriate keywords were combined using Boolean operators in order to develop the search input. A comprehensive list of search terms used for each database is located within the Appendix A (Table A1). We conducted a search of five databases, namely, the Cumulative Index to Nursing and Allied Health Literature (CINAHL), Excerpta Medica Database (EMBASE), PubMed, Scopus, and Web of Science, on 31 October 2023. To reflect the significant advancements of LLMs in recent years and identify emerging trends, we filtered out articles published prior to 2018. All identified articles were imported into EndNote software (Version 20.4.1) for reference management.

### 2.2. Study Eligibility and Selection Process

The database search resulted in the identification of 1179 articles. Subsequently, 528 duplicate records were removed. The screening of articles based on the title and abstract resulted in the further removal of 121 records deemed to be irrelevant. A subsequent eligibility assessment was performed by two independent reviewers (S.P. and S.B.) in order to identify studies that met the inclusion criteria. The inclusion criteria included surgery-related articles that focused on LLMs, such as their applications and limitations. Articles that were not focused on LLMs were excluded. Similarly, articles that were unrelated to surgery or had no intended surgery audience were excluded. We additionally excluded duplicate, unretrievable, non-English, and non-peer-reviewed articles. Furthermore, only articles with adequate ethical discussion were included. Articles with adequate ethical discussion were defined as having at least one paragraph dedicated to explicit ethical considerations or demonstrating adequate discussion throughout the article. An overview of eligibility criteria is located within the Appendix A (Table A2).

### 2.3. Data Collection and Analysis

Study details and the characteristics of ethical discussion were systematically extracted and organized using Microsoft Excel Version 2401 (Redmond, Washington). The extracted details included article type, surgical specialty, journal, discussed LLM, ethical principles, and ethical concerns. This information was then analyzed, summarized, and synthesized to offer a comprehensive overview of both the included articles and the ethical considerations discussed in these articles in the form of tables and graphs.

## 3. Results

### 3.1. Characteristics of Included Studies

**Figure 2:**
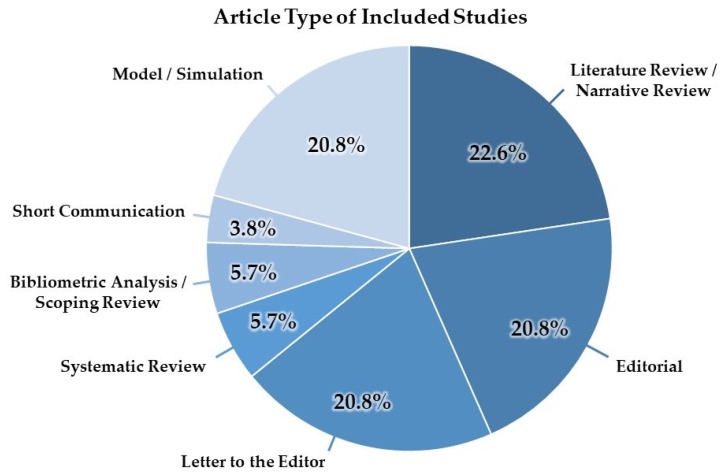
A pie chart depicting the article type of included studies.

The literature search yielded a total of 1179 results, of which 53 met the eligibility criteria. All included articles were published in 2023, and article types are displayed in Figure 2. Non-systematic reviews, such as literature and narrative reviews (n = 12), were the most common article type. Original studies simulated the capabilities of LLMs like ChatGPT to perform various tasks, such as diagnostic support. Plastic surgery (n = 14), orthopedic surgery (n = 9), and neurosurgery (n = 7) were the surgical specialties with the highest representation (Table 2). The included articles spanned 41 different journals. The *Aesthetic Surgery Journal* was the most represented, with five (9.4%) of the included articles published in this journal, followed by *Annals of Biomedical Engineering* (n = 3, 5.7%). OpenAI’s ChatGPT was mentioned in all (100%) of the included articles, with Google’s Bard as the next most frequently mentioned LLM (n = 11, 20.8%). In addition to OpenAI, other mentioned LLM developers included Google, Meta (formerly Facebook AI), and Microsoft, which were mentioned in 32.1%, 15.1%, and 9.4% of articles, respectively.

Given the qualitative nature of this review, which aims to explore ethical considerations within an emerging field, formal confidence analysis, heterogeneity assessment, and risk-of-bias tools were not applied to the included studies. Instead, we focused on synthesizing the diverse ethical themes and concerns identified within the literature.

### 3.2. Ethical Considerations

**Figure 3:**
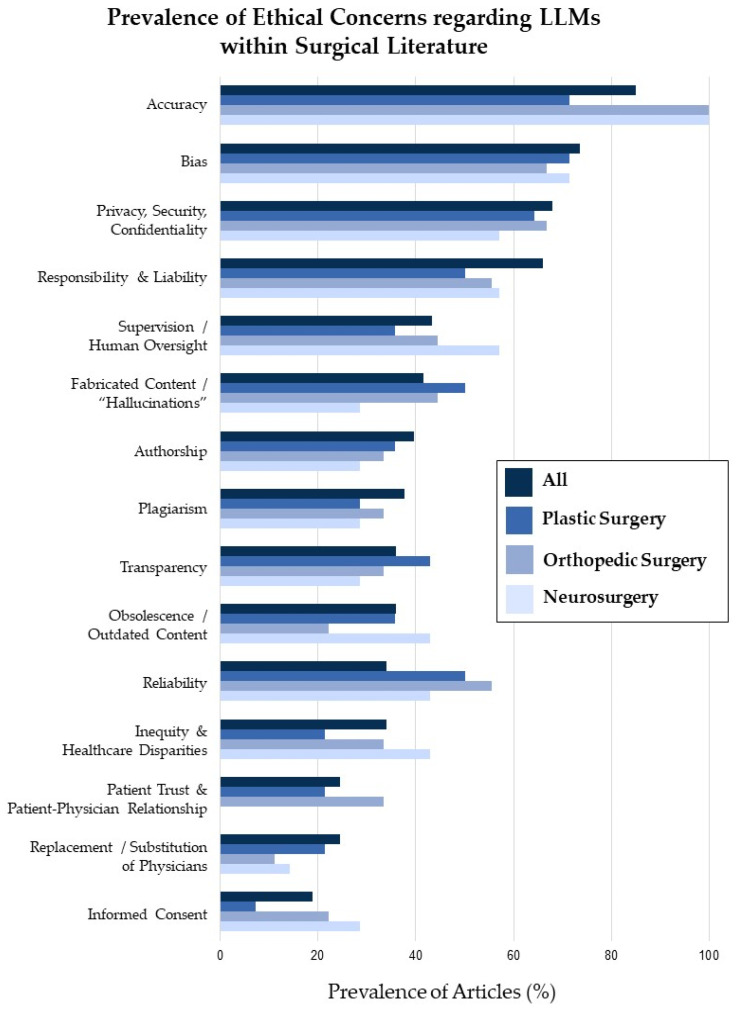
Graphical representation of the prevalence of ethical concerns regarding LLMs within surgery research as a whole (All) as well as within plastic surgery, orthopedic surgery, and neurosurgery literature.

The accuracy of LLMs and their content was the most cited concern, appearing in 45 (84.9%) of the included articles (Table 3). Accuracy, bias, patient confidentiality, and responsibility were not only the most cited ethical concerns overall, but the most frequently cited ethical concerns in plastic surgery, orthopedic surgery, and neurosurgery literature (Figure 3). Although concerns regarding reliability were mentioned in 18 (34%) articles overall, this concern was cited in 50.0%, 55.6%, and 42.9% of plastic surgery, neurosurgery, and orthopedic surgery articles, respectively. Although 13 (24.5%) of the articles overall mentioned patient trust and the patient-physician relationship, no neurosurgery articles expressed this concern. Considerations regarding transparency were notably higher in plastic surgery research, and these were cited in 42.9% of the studies compared to 28.6% of the neurosurgery articles. However, concerns regarding the need for supervision were more common in neurosurgery research (57.1%) compared to plastic surgery research (35.7%). Worries about fabricated content and “hallucinations” were cited less in neurosurgery research (28.6%) than in plastic surgery (50%), orthopedic surgery (44.4%), and across all articles (41.5%). Concerns regarding informed consent appeared in ten (18.9%) articles overall, but only in 7.1% of plastic surgery articles.

### 3.3. Representation of Ethical Principles

With explicit mention in ten (18.9%) of the included articles, autonomy was the most frequently mentioned ethical principle (Table 4). Nonmaleficence and justice were explicitly mentioned in four (7.5%) articles each. Beneficence was the least discussed principle, appearing in only three (5.7%) of the included articles, with these three articles explicitly naming all four ethical principles.

## 4. Discussion

**Figure 4:**
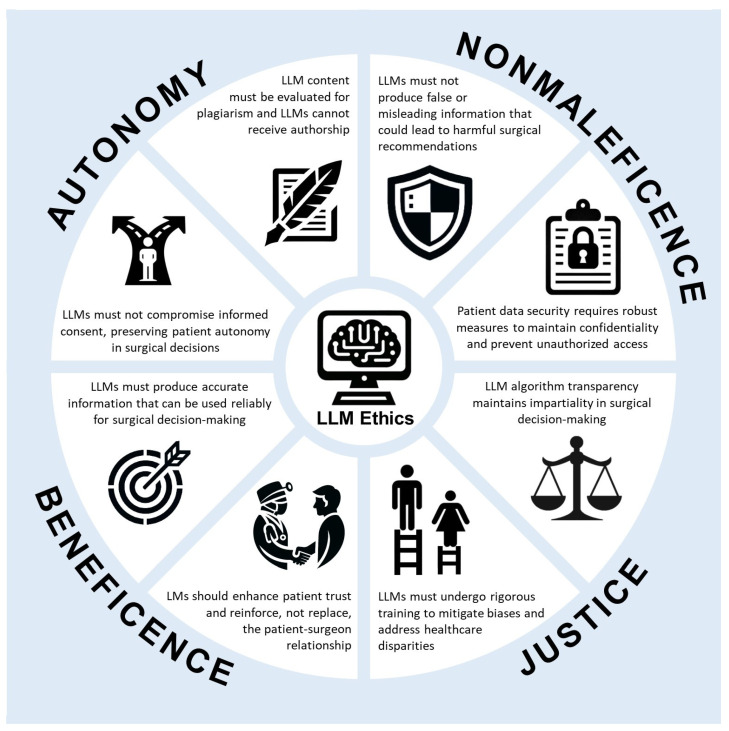
Infographic with examples of ethical concerns of LLMs and relevant ethical principles.

Many of the ethical concerns highlighted in the reviewed studies go beyond surgery and are pertinent to other medical specialties and healthcare as a whole (Figure 4). Concerns surrounding accuracy, bias, patient confidentiality, and responsibility are pervasive throughout healthcare practice, reflecting shared ethical dilemmas. This acknowledgment highlights the need for a unified approach to address ethical issues in healthcare, emphasizing collaboration and interdisciplinary dialogue to develop comprehensive solutions.

### 4.1. Representation of Ethical Principles

Ethical discussions in medical research are essential to ensuring that the pursuit of knowledge is balanced with respect for human dignity, social justice, and the broader public interest. However, ethical discourse is not always present in medical literature. Surgical literature was previously found to contain less ethical discourse than non-surgical medical research [22]. This trend was consistent with a 2009 systematic review by Chung et al., which found a paucity of ethical discussion in plastic surgery research, specifically. This review found that less than 1% of articles contained ethical discourse; of those that did, autonomy (53%) was the most frequently represented principle, and distributive justice (15%) was the least mentioned [29]. In a subsequent 2021 study by Chappell et al., who investigated ethical discourse in plastic surgery research, autonomy (87.9%) was the most commonly discussed principle, and justice (51.2%) was the least mentioned [30]. A similar 2022 study investigating ethical representation, specifically in COVID-19 research, found that nonmaleficence (53%) was the most frequently addressed principle [31]. However, autonomy (10%) was found to be the least represented principle, which contrasted previous studies.

In line with prior research on surgery literature, this review highlights autonomy as the most commonly referenced ethical principle, although we did not detect it as extensively as prior studies. The importance of autonomy is particularly pronounced in the surgical field. In the operating room, sedation and anesthesia render patients completely vulnerable. Because of this, surgery requires a strong level of communication and trust between surgeon and patient. Autonomy serves as the cornerstone of this relationship, ensuring that patients retain control over their decisions and are treated with respect, even in their most vulnerable state. However, less than a quarter of the studies analyzed in this review explicitly mentioned this important principle, with the majority failing to address ethical principles altogether. Nonetheless, the cited ethical concerns can still be examined from the lens of ethical principlism [21]. Additionally, future articles should strive to include ethical discussions that are grounded in established ethical frameworks like ethical principlism. Adhering to established ethical guidelines enhances research transparency by providing clear standards and frameworks for addressing ethical considerations in a structured and accountable manner. Furthermore, the use of ethical frameworks will help researchers to navigate ethical dilemmas, such as those associated with LLM use in surgical settings. 

### 4.2. Ethical Concerns

#### 4.2.1. Accuracy

In the studies included in this review, the most prevalent and commonly cited ethical concern surrounding LLMs is accuracy. Concerns regarding the accuracy, or rather inaccuracy, of content produced by LLMs are relevant to multiple ethical principles. Healthcare professionals, equipped with accurate and reliable information, are best positioned to make informed decisions and deliver evidence-based patient care that aligns with the highest standards of medical beneficence [21]. Furthermore, accurate medical information is essential in preventing patient harm, underscoring its relevance to the ethical principle of nonmaleficence. Inaccurate and unreliable information can lead to incorrect diagnosis, unnecessary testing, as well as ineffective and potentially harmful treatment [6,32,33,34]. LLMs have numerous potential applications in surgical contexts, but these are dependent on the accuracy and reliability of these models. For example, LLMs can support surgeons in preoperative diagnosis, analyzing patient data to identify surgical needs, and devising evidence-based plans to enhance outcomes and minimize risks [35,36]. These models can then assist in intraoperative decision making. LLMs can help to prepare surgical trainees by simulating procedures and offering real-time support and guidance regarding surgical techniques [6,35,36,37]. Following surgery, LLMs can provide personalized management recommendations and postoperative care instructions, with potential existing for remote patient monitoring [38,39]. Additionally, LLMs can draft consultation reports, patient encounters, surgical consent forms, operative notes, and discharge summaries [6,36,39]. While this assistance with documentation can streamline workflow, it requires complete accuracy.

Problems with inaccuracy can originate from inaccurate, incomplete, outdated, or even biased datasets [32]. Additionally, limitations in understanding complex or context-dependent information can also lead to suboptimal responses [40,41]. One article discussed how, since LLMs employ machine learning, they are susceptible to the phenomenon of drift [42]. This drift leads to a gradual decline in performance over time as the training data age and become less relevant [43]. Ultimately, physicians must verify the information generated by an LLM and ensure that the use of LLMs aligns with the best interests of the patient [40]. The responsible use of LLMs will require professional oversight by human medical professionals to ensure accuracy and reliability [15,36,44,45,46].

#### 4.2.2. Fabricated Content and “Hallucinations”

The ethical use of LLMs in medicine is significantly challenged by their tendency to generate fabricated responses [47,48] when lacking information [49]. This phenomenon of AI “hallucination” [6,32,34,41,44,47,48,49,50,51,52,53], previously described as “stochastic parroting” [19], directly challenges the ethical principle of nonmaleficence, which calls for the avoidance of patient harm. In healthcare, the implications of such inaccuracies can be profound, ranging from false diagnoses to the suggestion of nonexistent symptoms or treatment protocols, thereby risking patient safety [36,54,55,56]. While LLMs can help to guide surgical candidacies and recommend treatment plans rooted in evidence-based data analysis, there exists a concern regarding their reliability [35,36,57]. These systems may inadvertently generate guidelines based on hallucinated or fabricated evidence, leading to deviations from established medical practices [6]. Such deviations pose significant risks as they can potentially compromise patient safety and treatment efficacy. In surgical research, there are concerns that LLMs fabricate not only references [52,58,59,60], but also data and results [46,55,61,62]. In order to uphold medical ethics, it is crucial to ensure that LLMs are meticulously trained and equipped to recognize and circumvent the creation of false or misleading medical information. This is vital in order to safeguard the integrity of medical advice and patient care, ensuring that LLMs support rather than compromise the quality of healthcare delivery.

#### 4.2.3. Informed Consent

Informed consent is a fundamental aspect of ethical medical practice, particularly in efforts to respect patient autonomy [21,32]. A 2020 article exploring the explainability of AI discussed how informed consent requires the full disclosure of risks and benefits, and how not disclosing the involvement of an AI system can undermine patient autonomy, patient trust, and the patient-physician relationship [63]. This sentiment was shared by multiple studies included in this review, which argued that informed consent should include a comprehensive disclosure of LLM-related risks and benefits and should precede LLM use in patient care [15,35,38,39,59]. However, the inherent complexity of LLM technology poses a significant challenge in this context. It can be difficult for patients to fully grasp the extent and implications of LLM use, which might compromise the integrity of the informed consent process [32,37]. Therefore, the process of obtaining consent is not a one-time event but an ongoing dialogue, ensuring that patients remain informed and comfortable with the level of LLM involvement in their care. In addition to autonomy, informed consent supports the principle of nonmaleficence by ensuring that recommendations and care align with a patient’s values, minimizing patient harm [32].

Furthermore, beyond obtaining informed consent for LLM usage, there exists the notion of LLMs actively participating in the informed consent process. Informed consent is especially important in surgical specialties, which often involve irreversible procedures [25] that necessitate comprehensive discussions regarding the risks and benefits of such procedures [23,24]. Compared to other surgical specialties, informed consent was an underreported ethical concern in the plastic surgery literature included within this review. However, this was addressed in an article by Aljindan et al., who examined ChatGPT’s role in plastic surgery, showcasing its application in generating surgical consent forms [6]. Nevertheless, inaccuracies in the information provided by LLMs like ChatGPT regarding surgical risks and benefits can undermine the informed consent process. An additional progression beyond generating consent forms involves entrusting the informed consent process directly to LLMs. This was explored in an article by Allen et al., who specifically discussed the ethics behind delegating the informed consent process to AI [32]. The authors noted that the practice of delegating raises concerns about patient trust, privacy, accuracy, and responsibility, which will impact patient-physician relationships and treatment decisions. Ultimately, transparent communication is essential in order to maintain the integrity of the informed consent process when LLMs are involved.

#### 4.2.4. Patient Privacy and Data Security

Patient privacy and data security are commonly cited ethical concerns that are deeply intertwined with the principle of autonomy [59]. Ensuring confidentiality allows a patient to make informed decisions about their healthcare without fear of unauthorized access or exposure. In addition to respecting individual autonomy, confidentiality can prevent potential harm, aligning with the principle of nonmaleficence. Effective data protection is crucial in order to prevent traceability back to individual patients, thereby avoiding breaches that could lead to identity theft or other misuses of medical information [6,32,64]. Such breaches could result in physical, emotional, or financial damages, violating both the privacy and safety of patients [36,54,65,66,67]. While valid concerns about patient privacy and data security arise when patients share personal information or when conversational agents access patient data, these concerns are not exclusive to LLMs and apply more broadly to electronic patient record systems [32]. Here, in the United States, the Health Insurance Portability and Accountability Act (HIPAA) is already in place to protect patient confidentiality. Strict adherence to regulations like the HIPAA is essential in order to maintain privacy and safeguard data security, protecting the confidentiality of patient health information [15,37,39,40]. 

#### 4.2.5. Bias and Inequity

The ethical issue of bias and inequity in the use of LLMs in medicine is a significant concern, which is closely tied to the principle of justice [15]. This principle emphasizes fairness, equity, and impartiality in healthcare decisions and resource distribution [21]. Ensuring fairness and non-discrimination is one of the seven HLEG guidelines for developing trustworthy AI [20]. Inherent biases in training data can result in LLMs generating biased medical advice, leading to unfair or discriminatory treatment, and undermining the goal of equitable treatment [6,32,37,41,52,59,66,68]. Such biases are particularly problematic as they can exacerbate existing health disparities, disproportionately affecting minority and marginalized groups [33,36,51,56,60,64,69]. Utilizing biased LLMs in the selection of patients for surgery may compromise safety and efficacy by overlooking candidates who could benefit from surgery while subjecting others to unnecessary procedures instead of more suitable nonsurgical options. Mitigating biases in LLMs is crucial to upholding the ethical obligation of nonmaleficence, and thus ensuring fair and equitable healthcare outcomes for all. To address this challenge, it is crucial to develop LLMs by using diverse and inclusive datasets [37,39,60]. This approach helps to mitigate the risk of biased outcomes and ensures that the healthcare advice provided by these models is equitable for all patients [40]. The regular monitoring of LLM outputs is also essential in order to detect and correct any biases, maintaining the integrity of healthcare services [15,19,40]. Addressing bias in LLMs is not just a technical necessity but an ethical obligation, as it ensures that the advancements in AI and medicine contribute to reducing health disparities rather than exacerbating them. Furthermore, there can be unequal access in a way that exacerbates healthcare inequities due to factors like a lack of internet access [51] and the cost of LLM implementation [6,37,69,70]. The growing affordability and accessibility of LLMs [48] should prompt the development of initiatives to bring these AI advancements to underprivileged communities [38] in order to mitigate these healthcare disparities. 

#### 4.2.6. Transparency

In medical practice, transparency is a critical ethical concern when using LLMs. These sophisticated AI systems often function as “black boxes”, with decision-making processes that are not transparent to users or developers [32,48,51,55,57]. This opacity challenges the ethical principle of justice [21], which emphasizes fairness, equity, and impartiality in decision making and resource allocation. It also raises issues of accountability, bias, and broader ethical consequences, highlighting the need for these AI systems to be made more interpretable [19,46,51]. Transparency is not only crucial for the ethical principle of justice, but also in terms of upholding autonomy. Patients and healthcare professionals need to understand how LLMs derive their conclusions, including the limitations of these models, the data they are trained on, and their underlying algorithms [38,40,49]. This knowledge is essential for informed decision making in a healthcare setting, allowing both patients and providers to assess the reliability and appropriateness of the medical advice provided. Furthermore, it is ethically important for patients to be informed about, and possibly consent to, the use of LLMs in their treatment [40]. Considerations regarding transparency were notably higher in plastic surgery research compared to other surgical specialties. In plastic surgery, the higher emphasis on transparency may stem from the nature of aesthetic procedures and the need for comprehensive disclosure to patients [6,66]. These findings underscore the importance of uniformly promoting transparency across all surgical disciplines. Additionally, the disclosure of LLM use is critical for upholding the integrity of academic work and research [64,71]. For surgical practice and research, transparency is essential. The HLEG guidelines specifically include the need for transparency within their requirements for developing trustworthy AI [20]. The currently opaque nature of LLMs can lead to unclear conclusions, thereby risking uninformed or misguided medical decisions. Therefore, enhancing LLM transparency is not merely a technical requirement but a moral obligation, essential for sustaining trust and reliability in healthcare settings.

#### 4.2.7. Responsibility and Liability

The integration of LLMs into medicine introduces complex ethical questions regarding responsibility and liability, especially when their use contributes to medical mistakes [32,59,68]. These issues are fundamentally linked to the principle of justice [21], which involves the fair assignment of responsibility and accountability, along with equitable consequences for actions, in line with societal and legal standards. As LLMs increasingly participate in medical decision making, determining liability becomes a challenging task. When an LLM’s advice results in a medical error, leading to an adverse event, who is responsible [38,59,64,68,72]? Is it the developers who created the LLM, the medical professionals who depended on it, or the technology itself [37,67]? Although some sources argue that the physician should take responsibility [35,39,55,73], this ambiguity in assigning responsibility necessitates the establishment of clear guidelines and legal frameworks. The ethical principle of justice demands fair accountability for errors or negligence in medical care. In surgical settings, where precision and accountability are essential, clarity regarding responsibility and liability is vital in order to protect patient interests. Therefore, as LLMs become more prevalent in healthcare settings, it is crucial to develop comprehensive policies and legal structures that clearly define the roles and responsibilities of all parties involved.

#### 4.2.8. Authorship and Plagiarism

The use of LLMs in surgery research brings forth pressing ethical concerns regarding plagiarism and authorship. Researchers have the fundamental right to recognition and proper attribution for their work. Respect for authorship and proper crediting practices upholds the autonomy of researchers, ensuring that their intellectual property is acknowledged appropriately. However, LLMs present a unique challenge in this domain. They can produce content that could be mistaken for human-generated medical literature, raising significant issues about authorship [8,15,19,32,56,65,74,75]. However, LLMs like ChatGPT are ineligible for authorship due to their inability to accept responsibility [41,47,52,53,55,58,71,76]. Additionally, there is a risk that LLMs might inadvertently plagiarize content, leading to unintentional plagiarism by users [47,71,76]. Such scenarios can blur the lines of true authorship and violate intellectual property rights and academic integrity [47,76]. Furthermore, this undermines the principle of justice, which encourages transparency and accessibility in research practices. Proper attribution and plagiarism prevention ensure fairness, with the acknowledgement of all contributors and maintenance of integrity in research [77]. To address these concerns and uphold academic integrity, stringent measures and guidelines must be in place to safeguard against plagiarism and ensure proper authorship. Journals are already adding policies that restrict the extent of LLM use in scientific writing [78,79]. 

#### 4.2.9. Patient Trust and Patient-Physician Relationship

The use of LLMs in medicine has the potential to undermine the patient-physician relationship [15,35]. Furthermore, the implementation of LLMs can compromise patient trust [32,44]. These concerns relate to the ethical principles of autonomy and beneficence, particularly since beneficence is central to the patient-physician relationship [15]. The implementation of LLMs in healthcare could shift power dynamics, potentially diminishing a patient’s sense of priority and increasing their detachment from healthcare providers due to decreased face-to-face contact. This perception of impersonal care may lead to dissatisfaction and affect treatment adherence and outcomes [36]. Additionally, the sophisticated algorithms and cryptic operation of LLMs may also contribute to patient mistrust [72]. Furthermore, reliability concerns with LLMs may lead to patients being misinformed and doctor–patient conflicts [80]. This concern is particularly significant in surgical contexts due to the high stakes involved in surgical procedures. Maintaining a strong patient-surgeon relationship built on trust is crucial for ensuring patients feel confident and supported throughout the surgical process [36]. The erosion of trust can jeopardize the success of a procedure and overall patient outcomes. Ultimately, transparency is essential for building trust and maintaining the patient-physician relationship [38,40]. The implementation of LLMs as a tool, rather than a replacement, for clinicians aligns best with patient autonomy and the maintenance of trust [51].

#### 4.2.10. Replacement of Physicians

There are concerns that AI technology will eventually replace healthcare providers. As AI technology like LLMs progress and become more efficient at automating tasks, there are concerns this will result in an infringement on the human workforce [9,66]. This raises concerns regarding economic justice for displaced workers and the need for accountable, transparent decision making. LLMs can rapidly offer information and suggestions, but they are not a replacement for a physician’s knowledge and clinical judgment [36,38,40,60]. A lack of clinician supervision leaves patients vulnerable to receiving inaccurate and potentially harmful information. Therefore, oversight is essential [15]. LLMs should be viewed as complementary tools that can assist healthcare providers [6,19,41].

### 4.3. LLMs and Other AI Advancements in Surgery

The ethical concerns about using LLMs in healthcare must be understood within the broader landscape of innovation. Alongside LLMs, various other AI advancements are actively being explored in the surgical field, which promises to improve surgical education and patient care. Augmented reality (AR) and virtual reality (VR) technologies are already being investigated across multiple surgical specialties, offering potential benefits for medical education in areas of anatomy [81], surgical planning [82,83], and the implementation of surgical robotics [84,85]. Additionally, the surgical metaverse may one day play a role in the training of surgical trainees [86,87,88]. However, while these advancements hold immense promise, they also bring forth a new set of ethical challenges that demand careful investigation and consideration. As AR, VR, and the surgical metaverse continue to evolve, they are expected to encounter similar ethical concerns as LLMs, as well as novel challenges specific to their functionalities [89,90]. It is imperative to proactively address these ethical questions in order to ensure that technological innovations enhance patient care while upholding ethical principles and patient safety. Through the thorough investigation and thoughtful consideration, the healthcare community can navigate these challenges and harness the full potential of AI advancements in surgery and beyond.

### 4.4. Limitations of the Study

The authors anticipate that a large number of relevant articles will be published within this burgeoning field of research after their database search on 31 October 2023, and prior to the release of this review’s findings. However, there have been efforts to expedite this review’s release to address this limitation. While we extensively searched five databases, it is possible that high-quality, relevant studies from other sources were missed and, therefore, not included in this review. Additionally, the review focused on English-language articles, but non-English articles represented only 1% of the initially identified studies.

### 4.5. Steps for Future Research

The burgeoning field of LLMs in medicine presents exciting opportunities, but also raises complex ethical concerns. While this systematic review offered a glimpse into the current landscape of ethical considerations surrounding LLMs and their application in surgery, future research should delve deeper into several key areas:

1. Improving accuracy and reliability. Further LLM development is essential for improving accuracy. This will likely involve expanding training datasets to include reliable and comprehensive medical data. Additionally, continuous updates to these datasets are necessary to maintain alignment with the guidelines of current medical practice. Furthermore, the refinement of AI algorithms will also improve accuracy.
2. Quantifying and mitigating bias. Robust methods need to be developed and validated for detecting and quantifying bias in LLM outputs, particularly with respect to patient demographics, socioeconomic status, and medical conditions. Effective strategies must be explored and implemented to mitigate bias in both LLM training data and algorithms, ensuring equitable and non-discriminatory medical advice and decision making.
3. Transparency and explainability. Enhancing LLM transparency requires the development of interpretable AI frameworks that explain the reasoning behind LLM outputs and decisions. It is crucial to empower users in order to understand limitations and build trust. Investigating and adopting effective tools for visualizing and communicating the reasoning behind LLMs and their limitations to patients and healthcare professionals is essential in order to foster informed decision making and collaboration.
4. Responsibility and liability. Clear legal frameworks and guidelines that define responsibility and liability for LLM-related errors or adverse events in healthcare need to be established, thus ensuring accountability and fairness. In-depth research is needed on the legal and ethical implications of LLM implementation, with consideration paid to various stakeholder perspectives and the promotion of responsible development and deployment.
5. Patient trust and communication. Investigating the impact of LLM use on the patient-physician relationship is crucial in order to explore strategies for maintaining trust, open communication, and patient autonomy in a context of LLM involvement. Ethical guidelines and best practices for communication between healthcare professionals, patients, and LLMs need to be developed, thus fostering practices to ensure the maintenance of informed consent, shared decision making, and optimal patient care.
6. Long-term societal impact. Exploring the potential economic and social consequences of LLM integration in healthcare is critical. Concerns about job displacement and ensuring equitable access to AI-powered healthcare solutions need to be addressed. It is essential to conduct ethical impact assessments of LLM implementation, with consideration of broader societal implications and potential unintended consequences. It is also necessary to formulate responsible governance frameworks.

By actively addressing these research areas, we can ensure that LLMs are developed and employed ethically, responsibly, and for the benefit of all patients and healthcare stakeholders. As the field continues to evolve, ongoing research and collaboration will be crucial for harnessing the full potential of LLMs while mitigating potential harms and navigating the complex ethical landscape. 

## 5. Conclusions

The ethical considerations surrounding the use of LLMs in surgery and healthcare in general are multifaceted and evolving, underscoring the necessity for rigorous ethical discourse. Engaging in these ethical discussions not only cultivates transparency and accountability, but also sets the stage for improved research practices and enhanced patient outcomes. Central to these discussions are the ethical principles of autonomy, beneficence, nonmaleficence, and justice. Autonomy has prominence among these principles, reflecting the importance placed on respecting a patient’s right to make informed decisions regarding their surgical care.

Surveying the ethical concerns reported across the surgical literature, this review highlights accuracy as a prevalent ethical concern, with implications for patient care and safety. The ramifications of inaccuracies in LLM-driven diagnoses or treatment recommendations extend beyond mere errors; they encompass potential harm inflicted on patients through unnecessary surgical interventions or compromised outcomes. Additional significant ethical concerns include bias, patient confidentiality, and responsibility. These ethical concerns highlight specific areas in need of future research and attention. Enhancing LLM accuracy, reducing bias, and increasing transparency emerge as key starting points. The integration of these models into surgery necessitates continuous ethical discourse to ensure responsible and ethical use, balancing technological advancement with human dignity and safety. By addressing these ethical considerations, the surgical community can uphold ethical standards, foster trust, and promote equitable healthcare outcomes for all patients.

## Figures and Tables

**Table 1:** Ethical principles as defined by Beauchamp and Childress [21], with examples.

Principle	Definition [21]	Examples
Autonomy	Respect for an individual’s right to informed medical decision making	Informed consent; full disclosure and discussion regarding the risks and benefits of surgical intervention; full disclosure and discussion regarding the involvement of an LLM in a patient’s care; respecting a patient’s right to privacy and confidentiality; and respecting a researcher’s right to have proper recognition and attribution for their work.
Beneficence	Maximization of benefit to the patient (“do good”), while minimizing harm	Training of highly skilled, patient-focused surgeons; practice of evidence-based medicine; and supervision and verification to ensure LLM-generated content meets quality standards and is beneficial.
Nonmaleficence	Avoidance of patient harm (“do no harm”)	Ongoing efforts by surgeons to minimize surgical complications; the avoidance of unnecessary procedures; the identification and rectification of inaccurate, incomplete, or outdated information that can lead to potentially harmful recommendations; and stringent security measures to maintain patient confidentiality and prevent harmful repercussions from unauthorized disclosures or data breaches.
Justice	Fair distribution of healthcare resources	Conscious efforts to minimize bias that can widen healthcare disparities and inequalities; establishing infrastructure to support equitable resource allocation; and identifying and rectifying training dataset bias to avoid the production of biased content and recommendations.

**Table 2:** Characteristics and details of included studies.

Characteristic		No.	%
Surgical specialty			
	Plastic surgery	14	26.4
	Orthopedic surgery/foot and ankle surgery	9	17.0
	Neurosurgery	7	13.2
	Nonspecific/general surgery	4	7.5
	Urology	4	7.5
	Otolaryngology/oral and maxillofacial surgery	4	7.5
	Colorectal surgery	3	5.7
	Ophthalmology	3	5.7
	Surgical oncology	3	5.7
	Transplant surgery	2	3.8
	Pediatric surgery	1	1.9
	Obstetrics and gynecology	1	1.9
	Bariatric surgery	1	1.9
Cited Large Language Models (Developer)			
	ChatGPT (OpenAI)	53	100.0
	Bard (Google)	11	20.8
	LaMDA (Google)	5	9.4
	Bing Chat (Microsoft)	4	7.5
	PaLM/Med-PaLM-2 (Google)	3	5.7
	BERT (Google)	3	5.7
	T5 (Google)	3	5.7
	LLaMA (Meta)	3	5.7
	RoBERTa (Meta)	2	3.8
	Claude (Anthropic)	2	3.8

ChatGPT, Chat Generative Pre-Trained Transformer; LaMDA, Language Model for Dialogue Applications; PaLM, Pathways Language Model; BERT, Bidirectional Encoder Representations from Transformers; T5, Text-To-Text Transfer Transformer; LLaMA, Large Language Model Meta AI; RoBERTa, Robustly Optimized BERT Pretraining Approach.

**Table 3:** Most frequently cited ethical concerns.

Ethical Consideration	Most Relevant Ethical Principle(s)	No.	%
Accuracy	Beneficence, nonmaleficence	45	84.9
Bias	Justice, nonmaleficence	39	73.6
Privacy, security, confidentiality	Autonomy, nonmaleficence	36	67.9
Responsibility and liability	Justice	35	66.0
Supervision/human oversight	Beneficence, nonmaleficence	23	43.4
Fabricated content/“hallucinations”	Nonmaleficence	22	41.5
Authorship	Autonomy, justice	21	39.6
Plagiarism	Autonomy, justice	20	37.7
Obsolescence/outdated content	Nonmaleficence, beneficence	19	35.8
Transparency	Autonomy, justice	19	35.8
Inequity and healthcare disparities	Justice, nonmaleficence	18	34.0
Reliability	Beneficence, nonmaleficence	18	34.0
Replacement/substitution of physicians	Justice	13	24.5
Patient trust and patient-physician relationship	Autonomy, beneficence	13	24.5
Informed consent	Autonomy, nonmaleficence	10	18.9

**Table 4:** Explicit Mention of Ethical Principles.

Ethical Principle	No.	%
All	3	5.7
Autonomy	10	18.9
Nonmaleficence	4	7.5
Justice	4	7.5
Beneficence	3	5.7

The percentage does not equal 100 percent since not every article that discusses ethical considerations explicitly mentions ethical principles.

## Data Availability

The data that support the findings of this study are available from the corresponding author upon reasonable request.

## Abbreviations

AI	Artificial intelligence
BERT	Bidirectional Encoder Representations from Transformers
ChatGPT	Chat Generative Pre-Trained Transformer
CINAHL	Cumulative Index to Nursing and Allied Health Literature
EMBASE	Excerpta Medica Database
HIPAA	Health Insurance Portability and Accountability Act
HLEG	High-Level Expert Group
LaMDA	Language Model for Dialogue Applications
LLaMA	Large Language Model Meta AI
LLM	large language model
NLP	natural language processing
PaLM	Pathways Language Model
PRISMA	Preferred Reporting Items for Systematic Reviews and Meta-Analyses
RoBERTa	Robustly Optimized BERT Pretraining Approach
T5	Text-To-Text Transfer Transformer

## Appendix A

**Table A1:** Search Strategy.

Database	Search String
CINAHL (EBSCO)	((“large language model*”) OR (“language model*”) OR (“OpenAI”) OR (“GPT”) OR (“chatbot”) OR ((“Google”) AND (“PaLM”)) OR ((“Google”) AND (“BERT”)) OR ((“Anthropic”) AND (“Claude”)) OR ((“Meta”) AND (“LLaMA”)) OR ((“Stanford”) AND (“Alpaca”))) AND ((“plastic surger*”) OR (“reconstructive surger*”) OR (“aesthetic surger*”) OR (“cosmetic surg*”) OR (“pediatric surger*”) OR (“orthopedic surger*”) OR (“otolaryngology surger*”) OR (“ENT surger*”) OR (“head and neck surger*”) OR (“cardiothoracic surger*”) OR (“thoracic surger*”) OR (“oncologic surger*”) OR (“surgical oncology”) OR (“burn surger*”) OR (“endocrine surger*”) OR (“ophthalmic surger*”) OR (“gynecological surger*”) OR (“vascular surger*”) OR (“colorectal surger*”) OR (“trauma surger*”) OR (“oral and maxillofacial surger*”) OR (“oromaxillofacial surger*”) OR (“transplant surger*”) OR (“bariatric surger*”) OR (“hand surger*”) OR (“hepatobiliary surger*”) OR (“minimally invasive surger*”) OR (“fetal surger*”) OR (“robotic surger*”) OR (“urology”) OR (“urologic surger*”) OR (“neurologic surger*”) OR (“neurosurg*”) OR (“elbow surger*”) OR (“spine surger*”) OR (“craniofacial surger*”) OR (“podiatric surger*”) OR (“surgical critical care”) OR (“organ transplant”) OR (“obstetrics and gynecology”) OR (“obstetrics and gynaecology”) OR (“OBGYN”) OR (“OB/GYN”) OR (“OB GYN”) OR (“surgery”) OR (“surgical”) OR (“surgeries”) OR (“surgeon*”))
EMBASE (Ovid)	((“large language model*”) OR (“language model*”) OR (“OpenAI”) OR (“GPT”) OR (“chatbot”) OR ((“Google”) AND (“PaLM”)) OR ((“Google”) AND (“BERT”)) OR ((“Anthropic”) AND (“Claude”)) OR ((“Meta”) AND (“LLaMA”)) OR ((“Stanford”) AND (“Alpaca”))) AND ((“plastic surger*”) OR (“reconstructive surger*”) OR (“aesthetic surger*”) OR (“cosmetic surg*”) OR (“pediatric surger*”) OR (“orthopedic surger*”) OR (“otolaryngology surger*”) OR (“ENT surger*”) OR (“head and neck surger*”) OR (“cardiothoracic surger*”) OR (“thoracic surger*”) OR (“oncologic surger*”) OR (“surgical oncology”) OR (“burn surger*”) OR (“endocrine surger*”) OR (“ophthalmic surger*”) OR (“gynecological surger*”) OR (“vascular surger*”) OR (“colorectal surger*”) OR (“trauma surger*”) OR (“oral and maxillofacial surger*”) OR (“oromaxillofacial surger*”) OR (“transplant surger*”) OR (“bariatric surger*”) OR (“hand surger*”) OR (“hepatobiliary surger*”) OR (“minimally invasive surger*”) OR (“fetal surger*”) OR (“robotic surger*”) OR (“urology”) OR (“urologic surger*”) OR (“neurologic surger*”) OR (“neurosurg*”) OR (“elbow surger*”) OR (“spine surger*”) OR (“craniofacial surger*”) OR (“podiatric surger*”) OR (“surgical critical care”) OR (“organ transplant”) OR (“obstetrics and gynecology”) OR (“obstetrics and gynaecology”) OR (“OBGYN”) OR (“OB/GYN”) OR (“OB GYN”) OR (“surgery”) OR (“surgical”) OR (“surgeries”) OR (“surgeon*”))
PubMed (NIH)	((“large language model*”) OR (“language model*”) OR (“OpenAI”) OR (“GPT”) OR (“chatbot”) OR ((“Google”) AND (“PaLM”)) OR ((“Google”) AND (“BERT”)) OR ((“Anthropic”) AND (“Claude”)) OR ((“Meta”) AND (“LLaMA”)) OR ((“Stanford”) AND (“Alpaca”))) AND ((“plastic surger*”) OR (“reconstructive surger*”) OR (“aesthetic surger*”) OR (“cosmetic surg*”) OR (“pediatric surger*”) OR (“orthopedic surger*”) OR (“otolaryngology surger*”) OR (“ENT surger*”) OR (“head and neck surger*”) OR (“cardiothoracic surger*”) OR (“thoracic surger*”) OR (“oncologic surger*”) OR (“surgical oncology”) OR (“burn surger*”) OR (“endocrine surger*”) OR (“ophthalmic surger*”) OR (“gynecological surger*”) OR (“vascular surger*”) OR (“colorectal surger*”) OR (“trauma surger*”) OR (“oral and maxillofacial surger*”) OR (“oromaxillofacial surger*”) OR (“transplant surger*”) OR (“bariatric surger*”) OR (“hand surger*”) OR (“hepatobiliary surger*”) OR (“minimally invasive surger*”) OR (“fetal surger*”) OR (“robotic surger*”) OR (“urology”) OR (“urologic surger*”) OR (“neurologic surger*”) OR (“neurosurg*”) OR (“elbow surger*”) OR (“spine surger*”) OR (“craniofacial surger*”) OR (“podiatric surger*”) OR (“surgical critical care”) OR (“organ transplant”) OR (“obstetrics and gynecology”) OR (“obstetrics and gynaecology”) OR (“OBGYN”) OR (“OB/GYN”) OR (“OB GYN”) OR (“surgery”) OR (“surgical”) OR (“surgeries”) OR (“surgeon*”))
Scopus (Elsevier)	((“large language model*”) OR (“language model*”) OR (“OpenAI”) OR (“GPT”) OR (“chatbot”) OR ((“Google”) AND (“PaLM”)) OR ((“Google”) AND (“BERT”)) OR ((“Anthropic”) AND (“Claude”)) OR ((“Meta”) AND (“LLaMA”)) OR ((“Stanford”) AND (“Alpaca”))) AND ((“plastic surger*”) OR (“reconstructive surger*”) OR (“aesthetic surger*”) OR (“cosmetic surg*”) OR (“pediatric surger*”) OR (“orthopedic surger*”) OR (“otolaryngology surger*”) OR (“ENT surger*”) OR (“head and neck surger*”) OR (“cardiothoracic surger*”) OR (“thoracic surger*”) OR (“oncologic surger*”) OR (“surgical oncology”) OR (“burn surger*”) OR (“endocrine surger*”) OR (“ophthalmic surger*”) OR (“gynecological surger*”) OR (“vascular surger*”) OR (“colorectal surger*”) OR (“trauma surger*”) OR (“oral and maxillofacial surger*”) OR (“oromaxillofacial surger*”) OR (“transplant surger*”) OR (“bariatric surger*”) OR (“hand surger*”) OR (“hepatobiliary surger*”) OR (“minimally invasive surger*”) OR (“fetal surger*”) OR (“robotic surger*”) OR (“urology”) OR (“urologic surger*”) OR (“neurologic surger*”) OR (“neurosurg*”) OR (“elbow surger*”) OR (“spine surger*”) OR (“craniofacial surger*”) OR (“podiatric surger*”) OR (“surgical critical care”) OR (“organ transplant”) OR (“obstetrics and gynecology”) OR (“obstetrics and gynaecology”) OR (“OBGYN”) OR (“OB/GYN”) OR (“OB GYN”) OR (“surgery”) OR (“surgical”) OR (“surgeries”) OR (“surgeon*”))
Web of Science (Thompson)	((“large language model*”) OR (“language model*”) OR (“openmi”) OR (“GPT”) OR (“chatbot”) OR ((“Google”) AND (“PaLM”)) OR ((“Google”) AND (“BERT”)) OR ((“Anthropic”) AND (“Claude”)) OR ((“Meta”) AND (“LLaMA”)) OR ((“Stanford”) AND (“Alpaca”))) AND ((“plastic surger*”) OR (“reconstructive surger*”) OR (“aesthetic surger*”) OR (“cosmetic surg*”) OR (“pediatric surger*”) OR (“orthopedic surger*”) OR (“otolaryngology surger*”) OR (“ENT surger*”) OR (“head and neck surger*”) OR (“cardiothoracic surger*”) OR (“thoracic surger*”) OR (“oncologic surger*”) OR (“surgical oncology”) OR (“burn surger*”) OR (“endocrine surger*”) OR (“ophthalmic surger*”) OR (“gynecological surger*”) OR (“vascular surger*”) OR (“colorectal surger*”) OR (“trauma surger*”) OR (“oral and maxillofacial surger*”) OR (“oromaxillofacial surger*”) OR (“transplant surger*”) OR (“bariatric surger*”) OR (“hand surger*”) OR (“hepatobiliary surger*”) OR (“minimally invasive surger*”) OR (“fetal surger*”) OR (“robotic surger*”) OR (“urology”) OR (“urologic surger*”) OR (“neurologic surger*”) OR (“neurosurg*”) OR (“elbow surger*”) OR (“spine surger*”) OR (“craniofacial surger*”) OR (“podiatric surger*”) OR (“surgical critical care”) OR (“organ transplant”) OR (“obstetrics and gynecology”) OR (“obstetrics and gynaecology”) OR (“obgyns”) OR (“OB/GYN”) OR (“OB GYN”) OR (“surgery”) OR (“surgical”) OR (“surgeries”) OR (“surgeon*”)) (Title) or ((“large language model*”) OR (“language model*”) OR (“openmi”) OR (“GPT”) OR (“chatbot”) OR ((“Google”) AND (“PaLM”)) OR ((“Google”) AND (“BERT”)) OR ((“Anthropic”) AND (“Claude”)) OR ((“Meta”) AND (“LLaMA”)) OR ((“Stanford”) AND (“Alpaca”))) AND ((“plastic surger*”) OR (“reconstructive surger*”) OR (“aesthetic surger*”) OR (“cosmetic surg*”) OR (“pediatric surger*”) OR (“orthopedic surger*”) OR (“otolaryngology surger*”) OR (“ENT surger*”) OR (“head and neck surger*”) OR (“cardiothoracic surger*”) OR (“thoracic surger*”) OR (“oncologic surger*”) OR (“surgical oncology”) OR (“burn surger*”) OR (“endocrine surger*”) OR (“ophthalmic surger*”) OR (“gynecological surger*”) OR (“vascular surger*”) OR (“colorectal surger*”) OR (“trauma surger*”) OR (“oral and maxillofacial surger*”) OR (“oromaxillofacial surger*”) OR (“transplant surger*”) OR (“bariatric surger*”) OR (“hand surger*”) OR (“hepatobiliary surger*”) OR (“minimally invasive surger*”) OR (“fetal surger*”) OR (“robotic surger*”) OR (“urology”) OR (“urologic surger*”) OR (“neurologic surger*”) OR (“neurosurg*”) OR (“elbow surger*”) OR (“spine surger*”) OR (“craniofacial surger*”) OR (“podiatric surger*”) OR (“surgical critical care”) OR (“organ transplant”) OR (“obstetrics and gynecology”) OR (“obstetrics and gynaecology”) OR (“obgyns”) OR (“OB/GYN”) OR (“OB GYN”) OR (“surgery”) OR (“surgical”) OR (“surgeries”) OR (“surgeon*”)) (Abstract)

Asterisks (*) in the table represent wildcard characters used in the search strategy to broaden the search scope during the systematic review process.

**Table A2:** Eligibility Criteria.

Inclusion Criteria	Exclusion Criteria
Articles published between 1 January 2018, and current (31 October 2023).	Articles published before 1 January 2018.
Articles written in English.	Articles not written in English.
Articles published in a peer-reviewed medical/scientific journal.	Articles that are unpublished (pre-print), published in a non-peer-reviewed journal, or non-journal articles (meeting abstracts, conference proceedings, etc.).
Full-text journal articles.	Unretrievable articles.
Sufficient discussion of ethical limitations.	Insufficient discussion of ethics.
Articles targeted toward a surgery audience and/or published in a surgical journal.	Articles not targeted toward a surgery audience or published in a surgical journal.
Articles focused on LLMs.	Articles that are not focused on LLMs.
	Duplicate article.
